# IL-35-producing B cells in gastric cancer patients

**DOI:** 10.1097/MD.0000000000010710

**Published:** 2018-05-11

**Authors:** Ke Wang, Jianming Liu, Jiansheng Li

**Affiliations:** a Department of Gastroenterology, The First Affiliated Hospital of Zhengzhou University, Zhengzhou; b Department of Pharmacology, JiangXi Medical College, Shangrao, China.

**Keywords:** gastric cancer, IL-35-producing B cells, myeloid-derived suppressor cells, regulatory T

## Abstract

A significant characteristic of advanced gastric cancer (GC) is immune suppression, which can promote the progression of GC. Interleukin 35 (IL-35) is an immune-suppressing cytokine, and it is generally recognized that this cytokine is secreted by regulatory T (Treg) cells. Recently, studies have found that IL-35 can also be produced by B cells in mice. However, scientific studies reporting that IL-35 is secreted by B cells in humans, specifically in cancer patients, are very rare.

Blood samples were collected from 30 healthy controls (HCs) and 50 untreated GC patients, and IL-35-producing B cells in the peripheral blood were investigated. Moreover, Treg cells (CD4^+^CD25^high/+^CD127^low/−^), myeloid-derived suppressor cells (MDSCs) (CD14^+^HLA-DR^low/−^) and other lymphocyte subsets (CD3^+^, CD4^+^, CD8^+^ T cells, activated and memory CD4^+^ T cells, activated CD8^+^ T cells, CD14^+^ monocytes, and IL-10-producing B cells) were also examined.

IL-35-producing B cells were significantly upregulated in patients with advanced GC. Furthermore, the frequency of IL-35-producing B cells was positively correlated with the frequencies of Treg cells (CD4^+^CD25^high/+^CD127^low/−^), MDSCs (CD14^+^HLA-DR^low/−^), IL-10-producing B cells, and CD14^+^ monocytes in these GC patients.

In summary, the frequency of IL-35-producing B cells is significantly elevated in advanced GC; this outcome implies that this group of B cells may participate in GC progression.

## 1 Introduction

Gastric cancer (GC) is one of the most common cancers worldwide, as approximately 951,600 new cases and an estimated 723,100 deaths occurred in 2012.^[1]^ Today, the relationship of the tumor microenvironment and the immune response is highly correlated, but the mechanism has not been clarified. Nevertheless, most concerns focus on the role of conventional immunosuppressive cells, including regulatory T (Treg) cells, myeloid and monocytic sub-populations, the presence of which has been widely reported in tumor patients. However, accumulating evidence has recognized the role of B cells in modulating the immune response to tumors, which is very important but is a double-edged sword. On the one hand, B cells can prevent tumor development through antigen presentation and antibody generation. On the other hand, B cells can accelerate tumor progression through various mechanisms.^[2–4]^

Recently, a new subset of B cells, known as regulatory B cells (Bregs), has been identified; these have been widely investigated in autoimmune diseases, transplantation, and inflammation. Moreover, many researchers are beginning to redirect their attention to the role of Bregs in cancer.^[2,5]^ However, no definitive phenotype of Breg cells has been confirmed, and the signaling pathway involved in their regulatory function has not yet been elucidated.^[6]^ Furthermore, as has been reported, Breg cells have diverse regulatory functions in different diseases, which has presented us with a substantial challenge. Currently, human Breg phenotypes include transitional B cells (CD19^+^CD24^hi^CD38^hi^), B regulatory 1 cells (Br1) (CD19^+^CD25^+^CD71^+^CD73^−^), plasmablasts (CD19^+^CD27^int^CD38^+^), and B10 cells with the CD19^+^CD24^hi^CD27^+^ phenotype.^[5,7]^ According to these studies of Breg cells, the immune-suppressing function of B cells occurs primarily via IL-10 secretion. The inhibitory function of these different phenotypes of Breg cells is evaluated based on the inhibitory function of the cytokine IL-10. Moreover, another cytokine, Interleukin 35 (IL-35), which is also secreted by B cells, may also play a key role in the immune suppression function of Breg cells.

IL-35 (p35/Epstein-Barr virus-induced gene 3 [EBI3]), which is a member of the IL-12 family, is a heterodimeric inhibitory cytokine composed of the IL-12 subunit p35 and the IL-27 subunit EBI3. IL-35 is preferentially secreted by Treg cells, which can inhibit T cell proliferation through cell cycle arrest, the modulation of the differentiation of T cells and the expansion of Treg cells.^[8,9]^ Importantly, as recently reported, IL-35 can also be produced by regulatory B cells in mice, but studies on IL-35 secretion by B cells in humans are very scarce.^[10]^

Therefore, a thorough understanding of tumor-associated B-cell subsets and their effects on tumor progression is very important, as this can accelerate the development of new therapies; in addition, IL-35-producing B cells may become a potential prognostic biomarker for GC patients. Thus, in this study, IL-35-producing B cells in GC and their potential relationship with other immune cells were investigated.

## 2 Materials and methods

### 2.1 Patients

We collected peripheral blood samples from 50 untreated GC patients at the time of diagnosis (31 men and 19 women; median age, 58.2 years; range, 29–97 years) and 30 matched healthy volunteers (17 men and 13 women; median age, 42.3 years; range, 25–54 years). Among the 50 GC patients, 9, 15, 16, and 10 were classified according to the WHO staging criteria as stage I, II, III, and IV, respectively. This study was approved by the Review Board of the First Affiliated Hospital of Zhengzhou University. Additionally, written informed consent for their sample analysis was obtained from all patients. All these samples were routinely tested to exclude the presence of viral and bacterial infections. Furthermore, individuals with autoimmune diseases were also excluded.

### 2.2 Flow cytometry: surface antigen staining

Lymphocyte phenotypes were shown by 7-color surface staining using the following anti-human monoclonal antibodies (MoAbs): CD3-APC (Beckman Coulter, Brea, CA; Cat. IM2467U), CD4-FITC (Beckman Coulter, Cat. A07750), CD4-PC5 (Beckman Coulter, Cat. A07752), CD14-APC-Cy7 (BD Pharmingen, San Diego, CA; Cat. 333951), CD8-PE-Cy7 (BD Pharmingen, Cat. 335822), CD19-FITC (Beckman Coulter, Cat. A07768), CD25-PE (Beckman Coulter, Cat. A07774), CD38-APC (BD Pharmingen, Cat. 345807), CD45RA-FITC (Beckman Coulter, Cat. A07786), CD45RO-PE (Beckman Coulter, Cat. A0087), CD127-Percp-cy5.5 (BD Pharmingen, Cat. 560551), and HLA-DR-Percp-cy5.5 (Beckman Coulter, Cat. A07793). Cells that were stained with different antibodies were defined as Treg cells (CD4^+^CD25^high/+^CD127^low/−^) or myeloid derived suppressor cells (MDSCs) (CD14^+^HLA-DR^low/−^). CD4^+^HLA-DR^+^ cells were regarded as activated CD4^+^ T cells, CD4^+^CD45RA^+^ cells were regarded as naive CD4^+^ T cells, and CD4^+^CD45RO^+^ cells were regarded as memory CD4^+^ T cells. CD8^+^HLA-DR^+^ cells and CD8^+^CD38^+^ cells were both regarded as activated CD8^+^ T cells, while CD14^+^ cells were regarded as monocytes. Matched isotype controls were used. Peripheral blood mononuclear cells (PBMCs) were incubated with labeled monoclonal antibodies for 20 minutes in the dark at room temperature. Samples were measured in a Canto II flow cytometer (BD Biosciences; San Jose, CA) and a Navios flow cytometer (Beckman coulter), and the analysis was performed using FlowJo software, Ashland, OR.

### 2.3 Intracellular cytokine staining

PBMCs were isolated by standard Ficoll density gradient centrifugation (DAKEWE, Shenzhen, P.R. China). Then, the cells were resuspended in RPMI-1640 medium (Gibco, Invitrogen, Waltham, MA) supplemented with 10% heat inactivated fetal calf serum (Gibco, Invitrogen), 100 U/mL penicillin, and 100 mg/mL streptomycin (both Gibco, Invitrogen). The cells were cultured with 2 μL Leukocyte Activation Cocktail with BD GolgiPlug (BD Biosciences, Cat. 550583) for 5 hours. Next, the cells were stained with Zombie NIR Fixable Viability Kit (Biolegend, San Diego, CA; Cat. 423105) and incubated at room temperature in the dark for 15 minutes to identify viable cells. Then, the cells were fixed in BD Cytofix/Cytoperm (BD Biosciences, Cat. 554714). Finally, cell surface and intracellular staining was performed with CD19-FITC (Beckman), IL-12/IL-35 p35-PerCP (R&D Systems, Minneapolis, MN; Cat. IC2191C), and IL-27/IL35 EBI3-PE (Biolegend, Cat. 360904). Moreover, the procedure for IL-10 staining was the same as that described above, except that the intracellular staining was stained with IL-10-APC (BD Pharmingen, Cat. 506807) in the final step. Unstained PBMCs and isotype controls were used to confirm the specificity of staining. Forward-scatter-width (FSC-W) versus forward-scatter-area (FSC-A) gating allowed the exclusion of cell doublets. EBI3^+^p35^+^CD19^+^ cells were defined as IL-35-producing B cells.

### 2.4 Statistical analysis

Statistical analysis was performed using GraphPad Prism software, La Jolla, CA, and the Mann–Whitney *U* test and Spearman rank correlation were performed. In addition, quantitative data are presented as the mean values ± standard deviation (SD). Differences were considered statistically significant at values of ∗*P* < .05, ∗∗*P* < .01, and ∗∗∗*P* < .0001.

## 3 Results

### 3.1 Upregulation of the frequency of IL-35-producing B cells in GC patients

**Figure 1:**
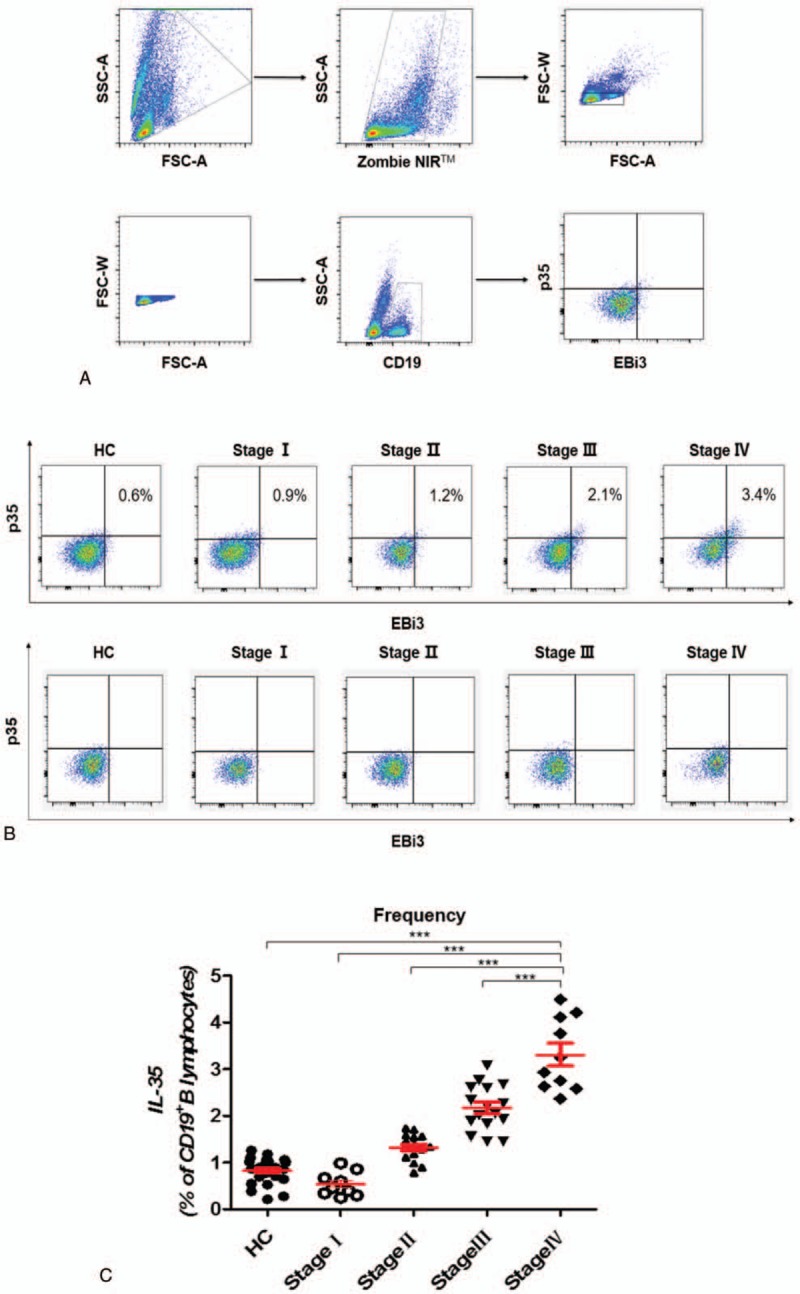
The population of IL-35-producing B cells was significantly expanded in GC patients. (A) Gating criteria from the peripheral blood for the live CD19^+^B cells and IL-35-producing B cells of GC patients are shown. (B) Representative data depicting IL-35-producing B cells in HCs and in patients with different stages of GC are shown with isotype. (C) The frequency of IL-35-producing B cells in patients with stage IV of GC (3.31 ± 0.24, n = 10) was obviously higher than the frequency in HCs (0.86 ± 0.05, n = 30, *P* < .0001), those with stage I disease (0.54 ± 0.09, n = 9, *P* < .0001), those with stage II disease (1.38 ± 0.08, n = 15, *P* < .0001), and those with stage III disease (2.22 ± 0.13, n = 16, *P* = .0002). The *P*-value was calculated by the Mann–Whitney *U* test. ∗∗∗*P* < .0001. GC = gastric cancer, HC = healthy control, IL-35 = Interleukin 35.

To investigate the frequency of IL-35-producing B cells in the peripheral blood of GC patients, we first gated B cells with the B cell surface marker CD19 and then used the EBI3^+^p35^+^ marker to identify IL-35-producing B cells (Fig. 1A). Second, we analyzed the expression of IL-35 in 50 untreated GC patients and 30 HCs and revealed that the frequency of IL-35-producing B cells was significantly elevated in GC patients, especially in those at an advanced stage (Fig. 1B). Furthermore, statistical analyses showed that the frequency of IL-35-producing B cells was positively associated with clinical progression (Fig. 1C), which implies that this subset of B cells may participate in the progression of GC.

### 3.2 The frequency of IL-35-producing B cells was significantly associated with the frequency of Treg cells (CD4^+^CD25^high/+^CD127^low/−^) and MDSCs (CD14^+^HLA-DR^low/−^) in GC patients

**Figure 2:**
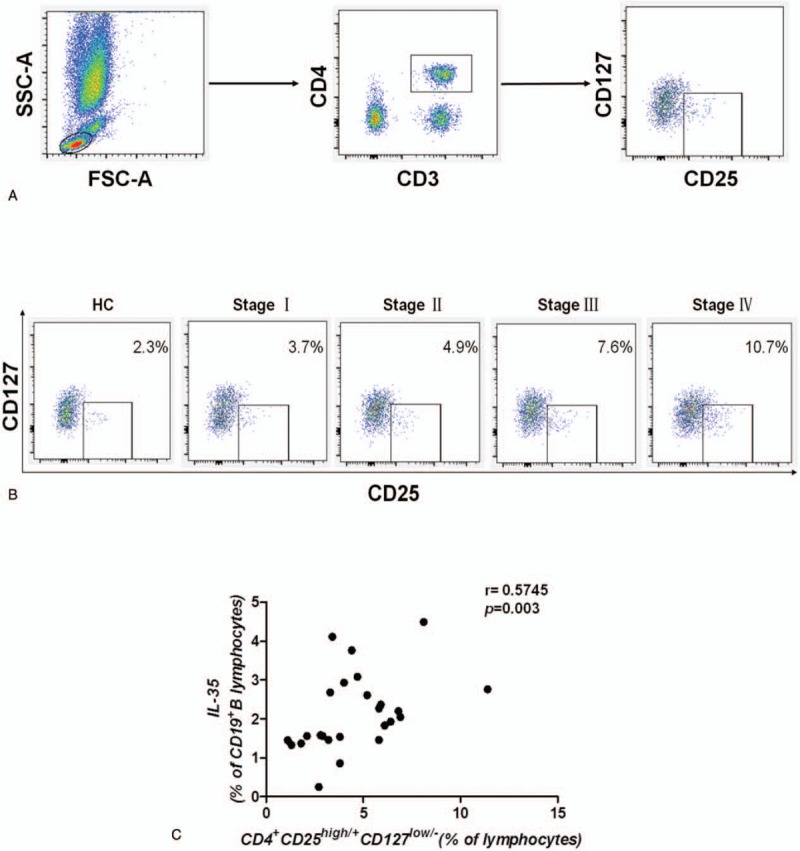
IL-35-producing B cells were associated with Treg cells in GC patients. (A) Gating criteria from the peripheral blood for the Treg (CD4^+^CD25^high/+^CD127^low/–^) cells of GC patients are shown. (B) Representative data depicting the ratio of Treg cells in patients with different stages of GC are shown. (C) The frequency of IL-35-producing B cells was positively correlated with the frequency of Treg cells (*r* = 0.5745, *P* = .0027, n = 25). A correlation analysis was performed using Spearman rank correlation. GC = gastric cancer, IL-35 = Interleukin 35, Treg cells = regulatory T cells.

**Figure 3:**
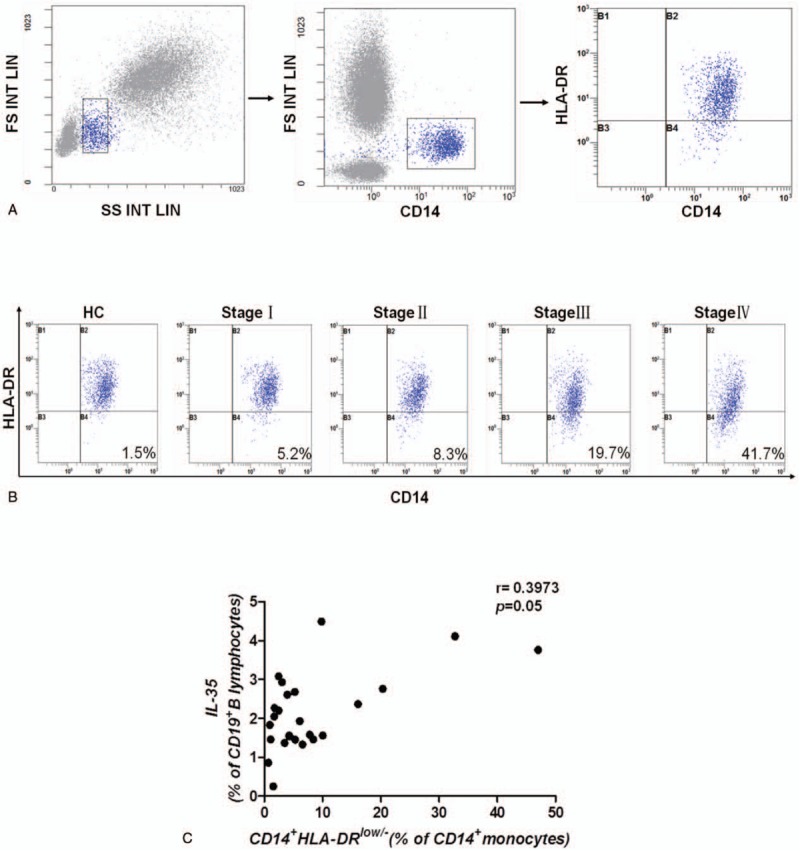
IL-35-producing B cells were associated with CD14^+^HLA-DR^low/–^ MDSCs in GC patients. Gating criteria from the peripheral blood for the CD14^+^HLA-DR^low/–^ MDSCs of GC patients are shown. (B) Representative data depicting the ratio of MDSCs in patients with different stages of GC are shown. (C) The frequency of IL-35-producing B cells was positively correlated with the frequency of CD14^+^HLA-DR^low/–^ MDSCs (*r* = 0.3973, *P* = .0492, n = 25). A correlation analysis was performed using Spearman rank correlation. GC = gastric cancer, IL-35 = Interleukin 35, MDSCs = myeloid-derived suppressor cells.

It is well known that Treg cells and MDSCs can suppress both innate and acquired immunity and can accelerate tumor progression. In our study, the frequency of various lymphocyte subsets was investigated. First, we gated Treg cells (Fig. 2A) and MDSCs (Fig. 3A), and then we found that the frequencies of Treg cells (Fig. 2B) and MDSCs (Fig. 3B) were also significantly elevated in GC patients. Further, the frequencies were positively associated with the clinical stage, similar to the frequency of IL-35-producing B cells. Spearman correlation analysis revealed that the frequency of IL-35-producing B cells was significantly positively correlated with the frequencies of Treg cells (CD4^+^CD25^high/+^CD127^low/−^) (*r* = 0.5745, *P* = .0027) (Fig. 2C) and MDSCs (CD14^+^HLA-DR^low/−^) (*r* = 0.3973, *P* = .0492) (Fig. 3C).

### 3.3 The frequency of IL-35-producing B cells was positively correlated with that of IL-10-producing B cells

**Figure 4:**
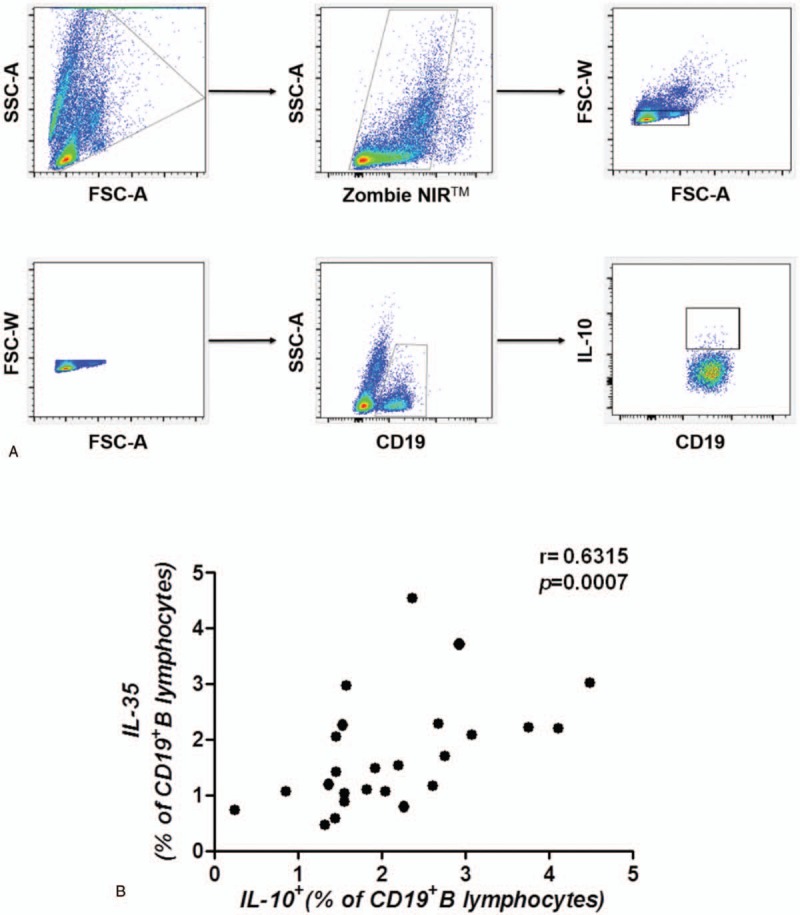
IL-35-producing B cells were associated IL-10-producing B cells. (A) Gating criteria from the peripheral blood for the live CD19^+^B cells and IL-10-producing B cells of GC patients are shown. (B) The frequency of IL-35-producing B cells was positively correlated with the frequency of IL-10-producing B cells (*r* = 0.6315, *P* = .0007, n = 25). A correlation analysis was performed using Spearman rank correlation. GC = gastric cancer, IL-35 = Interleukin 35.

It is known that IL-10, which is an immunosuppressive cytokine that can suppress the immune response, is currently used as a marker of regulatory B cells. In this study, we determined that the frequency of IL-10-producing B cells was positively correlated with the frequency of IL-35-producing B cells (*r* = 0.6315, *P* = .0007) (Fig. 4A, B), which indicates that B cells induce immunosuppression in GC patients potentially via both IL-10 and IL-35 secretion.

### 3.4 The frequency of IL-35-producing B cells demonstrated no correlation with other immune cell subsets but was positively correlated with the frequency of CD14^+^ monocytes

**Figure 5:**
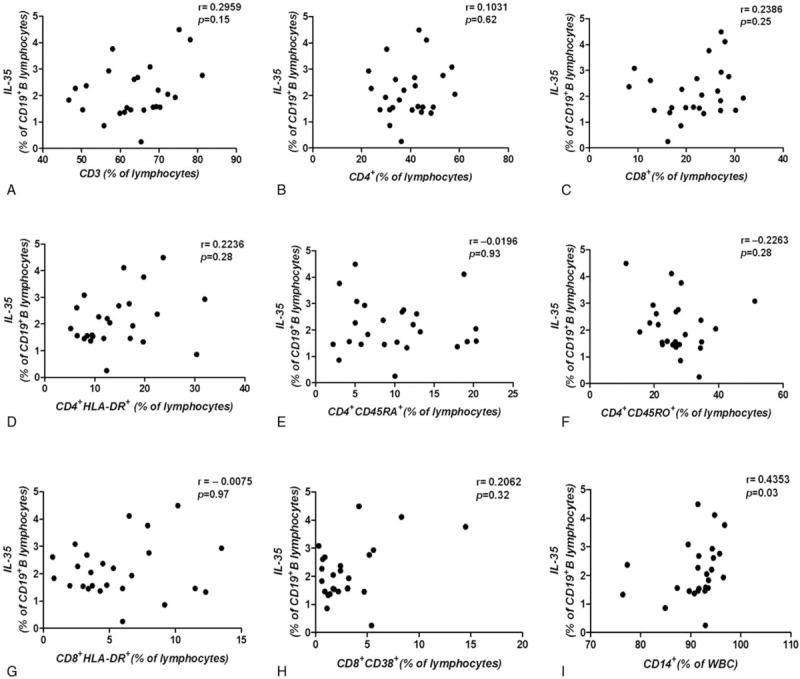
IL-35-producing B cells were associated with other immune cell subsets and CD14^+^ monocytes. (A, B, C) The frequency of IL-35-producing B cells was not correlated with the frequency of CD3^+^, CD4^+^, or CD8^+^ T cells (D, E, F) and was also not correlated with the frequency of activated CD4^+^ T cells and memory CD4^+^ T cells, and (G, H) with the frequency of activated CD8^+^ T cells. (I) The frequency of IL-35-producing B cells was positively correlated with the frequency CD14^+^ monocytes (*r* = 0.4353, *P* = .03, n = 25). A correlation analysis was performed using Spearman rank correlation. IL-35 = Interleukin 35.

The frequencies of CD3^+^, CD4^+^, CD8^+^, naive CD4^+^ T cells (CD4^+^CD45RA^+^ T cells), memory CD4^+^ T cells (CD4^+^CD45RO^+^ T cells), activated CD4^+^ T cells (CD4^+^HLA-DR^+^ T cells), and activated CD8^+^ T cells (CD8^+^HLA-DR^+^ or CD8^+^CD38^+^ T cells) were also assessed in this study. The results showed that the frequency of IL-35-producing B cells was not correlated with that of each immune cell subset mentioned above (Fig. 5A–H). However, the frequency of CD14^+^ monocytes was positively correlated with the frequency of IL-35-producing B cells (*r* = 0.4353, *P* = .0297) (Fig. 5I), which indicates the reciprocal interaction of monocytes and IL-35-producing B cells in GC patients in vivo.

## 4 Discussion

Thus far, many studies have found that Breg cells play an important role in malignant cancers, including hepatocellular carcinoma, esophageal cancer, ovarian cancer, tongue squamous cell carcinoma, and non-small cell lung carcinoma.^[11–16]^ All the above findings indicate that the immunosuppression induced by Breg cells is mainly mediated through IL-10 secretion. However, B cells not only have regulatory effects on IL-10 secretion but also generate IL-35, yet studies focusing on this phenomenon in cancer patients are very rare. Furthermore, IL-35 was initially reported as an inhibitory mediator secreted exclusively by Treg cells, and subsequently, Shen et al^[9,10]^ found that B cells are a novel source of IL-35.

Recently, various studies have demonstrated that IL-35 inhibits T cell proliferation through cell cycle arrest, the modulation of T cell differentiation and the expansion of Treg cells, but the relationship between IL-35 and B cells has not yet been elucidated.^[17–20]^ One study showed that IL-35 could induce the conversion of conventional B cells or IL-10-producing B cells into novel IL-35-producing B cells, which could also promote the expansion of IL-35-producing and IL-10-producing B cells.^[21]^ Another study found that EBI3 and p35 were highly expressed in tumor tissues and were positively correlated with tumor size. Similarly, IL-35 was also found to be highly expressed in GC, and thus, it might be involved in the growth of GC.^[22]^ Additionally, in pancreatic ductal adenocarcinoma samples from patients, the levels of IL-35 mRNA and protein were correlated with microvessel density and infiltration of monocyte lineage cells, which further supports our correlation study that found that the frequency of IL-35-producing B cells was correlated with that of CD14^+^ monocytes.^[23]^ Similarly, Wang et al^[24]^ also demonstrated that tumor-derived IL-35 promoted tumor growth by enhancing myeloid cell accumulation and angiogenesis. Furthermore, Zhao et al^[25]^ found that IL-35 expression was increased in tumor-infiltrating lymphocytes, which was correlated with poor prognosis in patients with breast cancer, but they did not distinguish which lymphocyte subset could produce the inhibitory cytokine IL-35.

In this study, we demonstrated that the frequency of IL-35-producing B cells was elevated in the peripheral blood of GC patients and that it was associated with the clinical stage. Moreover, IL-35-producing B cells were positively associated with Treg cells, MDSCs, IL-10-producing B cells, and CD14^+^ monocytes during the progression of GC, which indicates that the suppression of IL-35-producing B cells in GC may occur through interaction with other immunosuppressive cells, such as Treg cells and MDSCs, which participate in the immunosuppressive response in cancer.

The limitation of our study is that the analysis of IL-35 secretion by isolated B cells and the interaction between IL-35-producing B cells and other immunosuppressive cells were not studied. Therefore, it is not sufficiently reliable to assume that IL-35-producing B cells are actually involved in regulatory activity, and similarly, its mechanism also cannot be assumed. Additionally, many studies have revealed that IL-35 could suppress anti-tumor immunity via the inhibition of T cell differentiation in mice.^[26,27]^ However, in our study, the frequency of IL35-producing B cells was elevated in advanced stage GC, but no correlation was observed between the IL-35-producing B cells and the percentage of activated or effector T cells in lymphocytes. In fact, the number of activated or effector T cells did demonstrate a decreasing trend in patients with advanced stage GC, which was accompanied by an elevation of IL-35-producing B cells. However, the small sample size of this study may limit our ability to interpret the results of the correlation analysis. Furthermore, the lack of tissue samples may be another limitation, as we could not analyze the expression of IL-35-producing B cells in in situ tissues to support our results. Herein, our aim is to determine whether there is any association between IL-35-producing B cells and GC clinical stage.

In conclusion, this is the first report of a significantly elevated population of B cells that produce IL-35 in GC patients and its association with the clinical stage. Spearman correlation analysis revealed that the frequency of IL-35-producing B cells was positively correlated with the frequencies of Treg cells, MDSCs, IL-10^+^ B cells, and CD14^+^ monocytes in GC patients.

## Author contributions

Ke Wang performed experiments and analyzed the data; Jianming Liu helped perform the experiments. Jiansheng Li and Ke Wang designed the study and wrote the manuscript.

**Conceptualization:** Jiansheng Li.

**Data curation:** Ke Wang.

**Formal analysis:** Ke Wang.

**Investigation:** Ke Wang.

**Methodology:** Ke Wang.

**Software:** Ke Wang, Jianming Liu.

**Writing – original draft:** Ke Wang.

**Writing – review and editing:** Ke Wang.
